# Poly[bis­(*N*,*N*-dimethyl­acetamide)-1κ*O*,2κ*O*-bis­(μ_4_-thio­phene-2,5-di­car­boxyl­ato-1:2:1′:2′κ^4^ *O* ^2^:*O* ^2′^:*O* ^5^:*O* ^5′^)dizinc]

**DOI:** 10.1107/S160053681103981X

**Published:** 2011-10-05

**Authors:** Ming-Ming Du, Seik Weng Ng

**Affiliations:** a Yankuang Guohong Chemical Co. Ltd, Zoucheng 273500, People’s Republic of China; b Department of Chemistry, University of Malaya, 50603 Kuala Lumpur, Malaysia; c Chemistry Department, Faculty of Science, King Abdulaziz University, PO Box 80203 Jeddah, Saudi Arabia

## Abstract

In the title polymeric complex, [Zn_2_(C_6_H_2_O_4_S)_2_(C_4_H_9_NO)_2_]_*n*_, each carboxyl­ate group of the thio­phene-2,5-dicarboxyl­ate dianion bridges a pair of inversion-related dimethyl­acetamide-coordinated Zn^II^ atoms, generating a layer motif parallel to (101). The Zn^II^ atom shows a distorted square-pyramidal coordination; the apical site is occupied by the O atom of the dimethyl­acetamide mol­ecule, whereas the four basal sites are occupied by carboxyl­ate O atoms. In the crystal, the dimethyl­acetamide mol­ecule is disordered over two positions in a 0.72 (1):0.28 (1) ratio in respect of the C atoms.

## Related literature

For the 1,10-phenanthroline adduct of zinc 2,5-thio­phene­dicarboxyl­ate, see: Chen *et al.* (1999 ▶). For bond-length dimensions of the 2,5-thio­phene­dicarboxyl­ate ion, see: Wu *et al.* (2006 ▶).
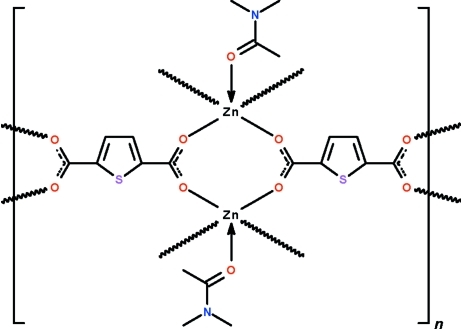


## Experimental

### 

#### Crystal data

[Zn_2_(C_6_H_2_O_4_S)_2_(C_4_H_9_NO)_2_]
*M* *_r_* = 645.26
Monoclinic, 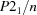

*a* = 8.4866 (2) Å
*b* = 14.8476 (4) Å
*c* = 10.1406 (3) Å
β = 100.734 (2)°
*V* = 1255.41 (6) Å^3^
*Z* = 2
Mo *K*α radiation
μ = 2.13 mm^−1^
*T* = 153 K
0.20 × 0.20 × 0.10 mm

#### Data collection

Gemini S Ultra diffractometer
Absorption correction: multi-scan (*CrysAlis PRO*; Agilent, 2010 ▶) *T*_min_ = 0.675, *T*_max_ = 0.815
7660 measured reflections
2841 independent reflections
1990 reflections with *I* > 2σ(*I*)
*R*_int_ = 0.029

#### Refinement

*R*[*F* ^2^ > 2σ(*F* ^2^)] = 0.034
*wR*(*F* ^2^) = 0.089
*S* = 0.93
2841 reflections
182 parameters
5 restraints
H-atom parameters constrained
Δρ_max_ = 1.13 e Å^−3^
Δρ_min_ = −0.55 e Å^−3^

### 

Data collection: *CrysAlis PRO* (Agilent, 2010 ▶); cell refinement: *CrysAlis PRO*; data reduction: *CrysAlis PRO*; program(s) used to solve structure: *SHELXS97* (Sheldrick, 2008 ▶); program(s) used to refine structure: *SHELXL97* (Sheldrick, 2008 ▶); molecular graphics: *X-SEED* (Barbour, 2001 ▶); software used to prepare material for publication: *publCIF* (Westrip, 2010 ▶).

## Supplementary Material

Crystal structure: contains datablock(s) global, I. DOI: 10.1107/S160053681103981X/xu5337sup1.cif

Structure factors: contains datablock(s) I. DOI: 10.1107/S160053681103981X/xu5337Isup2.hkl

Additional supplementary materials:  crystallographic information; 3D view; checkCIF report

## Figures and Tables

**Fig. 1.**
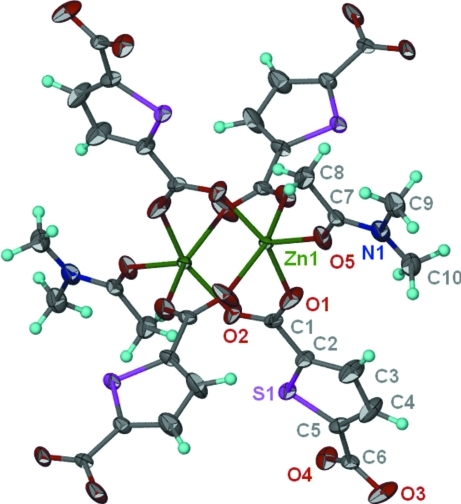
Thermal ellipsoid plot (Barbour, 2001) of a portion of polymeric Zn2(C4H9NO)2(C6H2O4S)2 at the 50% probability level; hydrogen atoms are drawn as spheres of arbitrary radius. The disorder in the dimethylacetamide unit is not shown. Only the asymmetric unit is labeled.

## supplementary crystallographic information

### Comment

The dianions of rigid aromatic carboxylic acids such as phthalic, isophthalic and terephathalic acids furnish coordination polymers with divalent metal ions. The 2,5-thiophenedicarboxylate anion is less well studied; the only crystal structure study of a zinc(II) system is that of the zinc 2,5-thiophenedicarboxylate adduct with 1,10-phenanthroline (Chen *et al.*, 1999). In this study, the dimethylacetamide (DMA) used as solvent in the synthesis is incorporate into the crystal structure. Polymeric [Zn_2_(C_4_H_9_NO)_2_(C_6_H_2_O_4_S)_2_]*_n_* (Scheme I) has the –CO_2_ parts of the thiophene-2,5-dicarboxylate dianion each bridging a pair of inversion-related, dimethylacetamide-coordinated zinc^II^ atoms to generate a layer motif (Fig. 1). The Zn^II^ atom shows square-pyramidal coordination; the apical site is occupied by the O atom of the DMA molecule. Bond dimensions involving the carboxylate unit are similar to those reported for ethylenediammonium thiophene-2,5-dicarboxylate dihydrate (Wu *et al.*, 2006).

### Experimental

2,5-Thiophenedicarboxylic acid (0.09 g, 0.5 mmol) and zinc nitrate hexahydrate (0.30 g,0.5 mmol) were dissolved in dimethylacetamide (10 ml). The solution was placed in a 25-ml flask, heated at 90 °C for 5 days. Colorless cystals separated from the solution upon cooling it to room temperature.

### Refinement

Carbon-bound H-atoms were placed in calculated positions [C—H 0.95 to 0.98 Å, *U*_iso_(H) 1.2 to 1.5*U*_eq_(C)] and were included in the refinement in the riding model approximation.

The DMA molecule is disordered over two positions with resect to the carbon atoms only. Pairs of distances (O–C, N–C) for the two components were restrained to within 0.01 Å of each other. The temperature factors of the primed atoms were set to those of the unprimed ones.

The final difference Fourier map had a peak at 1.04 Å from C3.

### Crystal data

**Table:**

[Zn_2_(C_6_H_2_O_4_S)_2_(C_4_H_9_NO)_2_]	*F*(000) = 656
*M**_r_* = 645.26	*D*_x_ = 1.707 Mg m^−^^3^
Monoclinic, *P*2_1_/*n*	Mo *K*α radiation, λ = 0.71073 Å
Hall symbol: -P 2yn	Cell parameters from 3158 reflections
*a* = 8.4866 (2) Å	θ = 2.4–29.7°
*b* = 14.8476 (4) Å	µ = 2.13 mm^−^^1^
*c* = 10.1406 (3) Å	*T* = 153 K
β = 100.734 (2)°	Block, colorless
*V* = 1255.41 (6) Å^3^	0.20 × 0.20 × 0.10 mm
*Z* = 2	

### Data collection

**Table:**

Gemini S Ultra diffractometer	2841 independent reflections
Radiation source: Enhance (Mo) X-ray Source	1990 reflections with *I* > 2σ(*I*)
graphite	*R*_int_ = 0.029
Detector resolution: 16.1903 pixels mm^-1^	θ_max_ = 27.5°, θ_min_ = 2.5°
ω scans	*h* = −10→11
Absorption correction: multi-scan (*CrysAlis PRO*; Agilent, 2010)	*k* = −19→17
*T*_min_ = 0.675, *T*_max_ = 0.815	*l* = −13→13
7660 measured reflections	

### Refinement

**Table:**

Refinement on *F*^2^	Primary atom site location: structure-invariant direct methods
Least-squares matrix: full	Secondary atom site location: difference Fourier map
*R*[*F*^2^ > 2σ(*F*^2^)] = 0.034	Hydrogen site location: inferred from neighbouring sites
*wR*(*F*^2^) = 0.089	H-atom parameters constrained
*S* = 0.93	*w* = 1/[σ^2^(*F*_o_^2^) + (0.0535*P*)^2^] where *P* = (*F*_o_^2^ + 2*F*_c_^2^)/3
2841 reflections	(Δ/σ)_max_ = 0.001
182 parameters	Δρ_max_ = 1.13 e Å^−^^3^
5 restraints	Δρ_min_ = −0.55 e Å^−^^3^

### Fractional atomic coordinates and isotropic or equivalent isotropic displacement parameters (Å^2^)

**Table:**

	*x*	*y*	*z*	*U*_iso_*/*U*_eq_	Occ. (<1)
Zn1	0.64210 (4)	0.53115 (2)	0.60386 (3)	0.02026 (12)	
S1	0.70730 (9)	0.23894 (5)	0.23932 (7)	0.02835 (19)	
O1	0.7270 (3)	0.42738 (18)	0.5087 (2)	0.0482 (7)	
O2	0.5233 (3)	0.38003 (16)	0.3549 (2)	0.0392 (6)	
O3	1.0411 (4)	0.06503 (19)	0.2061 (2)	0.0544 (8)	
O4	0.8306 (3)	0.10976 (17)	0.0555 (2)	0.0432 (6)	
O5	0.8383 (3)	0.55742 (18)	0.7354 (2)	0.0428 (6)	
N1	1.0425 (3)	0.5890 (2)	0.8985 (3)	0.0387 (7)	
C1	0.6643 (4)	0.3774 (2)	0.4147 (3)	0.0262 (7)	
C2	0.7708 (4)	0.3090 (2)	0.3709 (3)	0.0284 (7)	
C3	0.9309 (5)	0.2970 (3)	0.4216 (4)	0.0507 (10)	
H3	0.9908	0.3328	0.4909	0.061*	
C4	0.9958 (5)	0.2253 (3)	0.3589 (4)	0.0550 (12)	
H4	1.1043	0.2064	0.3830	0.066*	
C5	0.8871 (4)	0.1853 (2)	0.2597 (3)	0.0312 (7)	
C6	0.9207 (4)	0.1141 (2)	0.1669 (3)	0.0299 (7)	
C7	0.8881 (5)	0.6019 (3)	0.8283 (4)	0.0298 (11)	0.72 (1)
C8	0.7793 (6)	0.6729 (4)	0.8697 (5)	0.0425 (13)	0.72 (1)
H8A	0.6843	0.6800	0.7990	0.064*	0.72 (1)
H8B	0.8368	0.7303	0.8839	0.064*	0.72 (1)
H8C	0.7461	0.6544	0.9532	0.064*	0.72 (1)
C9	1.0963 (7)	0.6473 (4)	1.0207 (5)	0.0471 (14)	0.72 (1)
H9A	1.1523	0.7004	0.9953	0.071*	0.72 (1)
H9B	1.1691	0.6128	1.0887	0.071*	0.72 (1)
H9C	1.0027	0.6663	1.0573	0.071*	0.72 (1)
C10	1.1457 (6)	0.5301 (4)	0.8593 (6)	0.0488 (14)	0.72 (1)
H10A	1.2457	0.5612	0.8526	0.073*	0.72 (1)
H10B	1.0980	0.5049	0.7716	0.073*	0.72 (1)
H10C	1.1683	0.4813	0.9253	0.073*	0.72 (1)
C7'	0.9677 (10)	0.5478 (7)	0.7833 (9)	0.0298 (11)	0.28
C8'	1.0369 (16)	0.4825 (8)	0.6974 (13)	0.0425 (13)	0.28
H8'1	0.9955	0.4220	0.7093	0.064*	0.279 (6)
H8'2	1.1541	0.4823	0.7234	0.064*	0.279 (6)
H8'3	1.0065	0.5004	0.6030	0.064*	0.279 (6)
C9'	1.2159 (12)	0.5540 (10)	0.9429 (15)	0.0471 (14)	0.28
H9'1	1.2414	0.5502	1.0411	0.071*	0.279 (6)
H9'2	1.2907	0.5955	0.9114	0.071*	0.279 (6)
H9'3	1.2254	0.4941	0.9044	0.071*	0.279 (6)
C10'	0.9967 (18)	0.6518 (8)	0.9728 (14)	0.0488 (14)	0.28
H10D	1.0135	0.6314	1.0663	0.073*	0.279 (6)
H10E	0.8827	0.6648	0.9412	0.073*	0.279 (6)
H10F	1.0597	0.7066	0.9669	0.073*	0.279 (6)

### Atomic displacement parameters (Å^2^)

**Table:**

	*U*^11^	*U*^22^	*U*^33^	*U*^12^	*U*^13^	*U*^23^
Zn1	0.02204 (18)	0.01473 (18)	0.02585 (19)	−0.00035 (14)	0.00927 (13)	0.00035 (14)
S1	0.0292 (4)	0.0271 (4)	0.0297 (4)	0.0037 (3)	0.0078 (3)	−0.0091 (3)
O1	0.0437 (16)	0.0457 (16)	0.0504 (15)	0.0201 (12)	−0.0037 (12)	−0.0284 (13)
O2	0.0275 (13)	0.0387 (14)	0.0508 (14)	0.0083 (10)	0.0055 (10)	−0.0228 (12)
O3	0.071 (2)	0.0465 (16)	0.0451 (15)	0.0326 (15)	0.0095 (13)	−0.0162 (13)
O4	0.0420 (15)	0.0471 (16)	0.0420 (14)	0.0071 (12)	0.0123 (11)	−0.0218 (12)
O5	0.0324 (14)	0.0489 (16)	0.0444 (14)	−0.0157 (12)	−0.0001 (11)	0.0031 (13)
N1	0.0342 (17)	0.0409 (18)	0.0382 (16)	−0.0090 (14)	−0.0002 (13)	0.0064 (14)
C1	0.0343 (18)	0.0199 (16)	0.0261 (16)	0.0037 (13)	0.0102 (13)	0.0001 (13)
C2	0.0320 (18)	0.0264 (17)	0.0258 (15)	0.0058 (13)	0.0030 (13)	−0.0080 (13)
C3	0.052 (3)	0.052 (2)	0.043 (2)	0.018 (2)	−0.0052 (17)	−0.0188 (19)
C4	0.043 (2)	0.059 (3)	0.056 (2)	0.025 (2)	−0.0071 (19)	−0.021 (2)
C5	0.0386 (19)	0.0244 (17)	0.0318 (16)	0.0068 (14)	0.0094 (13)	−0.0052 (14)
C6	0.0369 (19)	0.0190 (17)	0.0393 (19)	0.0001 (14)	0.0215 (15)	−0.0049 (14)
C7	0.031 (2)	0.025 (2)	0.036 (2)	−0.0063 (17)	0.0121 (18)	0.0012 (18)
C8	0.036 (3)	0.044 (3)	0.047 (3)	0.003 (2)	0.007 (2)	−0.016 (2)
C9	0.040 (3)	0.057 (4)	0.042 (3)	−0.010 (2)	0.002 (2)	0.006 (2)
C10	0.036 (3)	0.052 (3)	0.059 (3)	−0.003 (2)	0.010 (2)	0.012 (3)
C7'	0.031 (2)	0.025 (2)	0.036 (2)	−0.0063 (17)	0.0121 (18)	0.0012 (18)
C8'	0.036 (3)	0.044 (3)	0.047 (3)	0.003 (2)	0.007 (2)	−0.016 (2)
C9'	0.040 (3)	0.057 (4)	0.042 (3)	−0.010 (2)	0.002 (2)	0.006 (2)
C10'	0.036 (3)	0.052 (3)	0.059 (3)	−0.003 (2)	0.010 (2)	0.012 (3)

### Geometric parameters (Å, °)

**Table:**

Zn1—O5	1.969 (2)	C3—C4	1.404 (5)
Zn1—O1	2.021 (2)	C3—H3	0.9500
Zn1—O2^i^	2.026 (2)	C4—C5	1.367 (5)
Zn1—O4^ii^	2.041 (2)	C4—H4	0.9500
Zn1—O3^iii^	2.044 (2)	C5—C6	1.479 (4)
Zn1—Zn1^i^	3.0360 (6)	C7—C8	1.511 (6)
S1—C2	1.699 (3)	C8—H8A	0.9800
S1—C5	1.699 (3)	C8—H8B	0.9800
O1—C1	1.246 (4)	C8—H8C	0.9800
O2—C1	1.237 (4)	C9—H9A	0.9800
O2—Zn1^i^	2.026 (2)	C9—H9B	0.9800
O3—C6	1.257 (4)	C9—H9C	0.9800
O3—Zn1^iv^	2.044 (2)	C10—H10A	0.9800
O4—C6	1.242 (4)	C10—H10B	0.9800
O4—Zn1^v^	2.041 (2)	C10—H10C	0.9800
O5—C7'	1.125 (8)	C7'—C8'	1.495 (10)
O5—C7	1.164 (4)	C8'—H8'1	0.9800
N1—C10'	1.303 (10)	C8'—H8'2	0.9800
N1—C10	1.349 (6)	C8'—H8'3	0.9800
N1—C7'	1.366 (8)	C9'—H9'1	0.9800
N1—C7	1.383 (5)	C9'—H9'2	0.9800
N1—C9	1.510 (5)	C9'—H9'3	0.9800
N1—C9'	1.547 (9)	C10'—H10D	0.9800
C1—C2	1.481 (4)	C10'—H10E	0.9800
C2—C3	1.372 (5)	C10'—H10F	0.9800
			
O5—Zn1—O1	98.22 (11)	C4—C3—H3	124.0
O5—Zn1—O2^i^	105.20 (10)	C5—C4—C3	113.3 (3)
O1—Zn1—O2^i^	156.55 (10)	C5—C4—H4	123.4
O5—Zn1—O4^ii^	102.52 (10)	C3—C4—H4	123.4
O1—Zn1—O4^ii^	87.40 (12)	C4—C5—C6	126.5 (3)
O2^i^—Zn1—O4^ii^	88.65 (11)	C4—C5—S1	110.7 (2)
O5—Zn1—O3^iii^	100.14 (10)	C6—C5—S1	122.2 (2)
O1—Zn1—O3^iii^	85.99 (13)	O4—C6—O3	125.8 (3)
O2^i^—Zn1—O3^iii^	88.75 (12)	O4—C6—C5	117.2 (3)
O4^ii^—Zn1—O3^iii^	157.07 (10)	O3—C6—C5	117.0 (3)
O5—Zn1—Zn1^i^	173.08 (8)	O5—C7—N1	120.3 (4)
O1—Zn1—Zn1^i^	75.18 (7)	O5—C7—C8	118.2 (4)
O2^i^—Zn1—Zn1^i^	81.37 (6)	N1—C7—C8	121.5 (4)
O4^ii^—Zn1—Zn1^i^	79.50 (7)	N1—C9—H9A	109.5
O3^iii^—Zn1—Zn1^i^	77.59 (7)	N1—C9—H9B	109.5
C2—S1—C5	92.59 (15)	N1—C9—H9C	109.5
C1—O1—Zn1	132.8 (2)	N1—C10—H10A	109.5
C1—O2—Zn1^i^	124.3 (2)	N1—C10—H10B	109.5
C6—O3—Zn1^iv^	129.6 (2)	N1—C10—H10C	109.5
C6—O4—Zn1^v^	127.4 (2)	O5—C7'—N1	125.0 (7)
C7'—O5—C7	62.9 (5)	O5—C7'—C8'	106.8 (7)
C7'—O5—Zn1	154.4 (5)	N1—C7'—C8'	128.2 (8)
C7—O5—Zn1	142.7 (3)	C7'—C8'—H8'1	109.5
C10'—N1—C10	156.1 (8)	C7'—C8'—H8'2	109.5
C10'—N1—C7'	132.4 (8)	H8'1—C8'—H8'2	109.5
C10—N1—C7'	71.4 (5)	C7'—C8'—H8'3	109.5
C10'—N1—C7	81.0 (7)	H8'1—C8'—H8'3	109.5
C10—N1—C7	122.9 (4)	H8'2—C8'—H8'3	109.5
C10—N1—C9	119.9 (4)	N1—C9'—H9'1	109.5
C7—N1—C9	117.1 (4)	N1—C9'—H9'2	109.5
C10'—N1—C9'	116.2 (9)	H9'1—C9'—H9'2	109.5
C7'—N1—C9'	111.3 (7)	N1—C9'—H9'3	109.5
O2—C1—O1	126.3 (3)	H9'1—C9'—H9'3	109.5
O2—C1—C2	117.6 (3)	H9'2—C9'—H9'3	109.5
O1—C1—C2	116.1 (3)	N1—C10'—H10D	109.5
C3—C2—C1	126.5 (3)	N1—C10'—H10E	109.5
C3—C2—S1	111.2 (2)	H10D—C10'—H10E	109.5
C1—C2—S1	122.2 (2)	N1—C10'—H10F	109.5
C2—C3—C4	111.9 (3)	H10D—C10'—H10F	109.5
C2—C3—H3	124.0	H10E—C10'—H10F	109.5
			
O5—Zn1—O1—C1	178.6 (3)	C4—C5—C6—O4	−152.9 (4)
O2^i^—Zn1—O1—C1	1.5 (5)	S1—C5—C6—O4	17.8 (4)
O4^ii^—Zn1—O1—C1	−79.1 (3)	C4—C5—C6—O3	24.9 (5)
O3^iii^—Zn1—O1—C1	78.9 (3)	S1—C5—C6—O3	−164.4 (3)
O1—Zn1—O5—C7'	2.8 (14)	C7'—O5—C7—N1	−5.0 (7)
O2^i^—Zn1—O5—C7'	−178.3 (14)	Zn1—O5—C7—N1	173.8 (3)
O4^ii^—Zn1—O5—C7'	−86.3 (14)	C7'—O5—C7—C8	175.6 (8)
O3^iii^—Zn1—O5—C7'	90.2 (14)	Zn1—O5—C7—C8	−5.7 (7)
O1—Zn1—O5—C7	−174.5 (4)	C10'—N1—C7—O5	−176.9 (7)
O2^i^—Zn1—O5—C7	4.3 (5)	C10—N1—C7—O5	4.7 (6)
O4^ii^—Zn1—O5—C7	96.3 (4)	C7'—N1—C7—O5	4.7 (6)
O3^iii^—Zn1—O5—C7	−87.2 (5)	C9—N1—C7—O5	−177.9 (4)
Zn1^i^—O2—C1—O1	0.4 (5)	C9'—N1—C7—O5	4(2)
Zn1^i^—O2—C1—C2	−179.1 (2)	C10'—N1—C7—C8	2.5 (8)
Zn1—O1—C1—O2	−1.0 (5)	C10—N1—C7—C8	−175.9 (5)
Zn1—O1—C1—C2	178.6 (2)	C7'—N1—C7—C8	−175.9 (8)
O2—C1—C2—C3	178.1 (4)	C9—N1—C7—C8	1.5 (6)
O1—C1—C2—C3	−1.4 (5)	C9'—N1—C7—C8	−176 (2)
O2—C1—C2—S1	2.6 (4)	C7—O5—C7'—N1	5.3 (7)
O1—C1—C2—S1	−176.9 (3)	Zn1—O5—C7'—N1	−172.9 (4)
C5—S1—C2—C3	5.4 (3)	C7—O5—C7'—C8'	−174.9 (11)
C5—S1—C2—C1	−178.5 (3)	Zn1—O5—C7'—C8'	7(2)
C1—C2—C3—C4	179.1 (4)	C10'—N1—C7'—O5	−7.2 (17)
S1—C2—C3—C4	−5.0 (5)	C10—N1—C7'—O5	175.0 (12)
C2—C3—C4—C5	1.7 (6)	C7—N1—C7'—O5	−5.1 (7)
C3—C4—C5—C6	174.0 (4)	C9—N1—C7'—O5	−16 (4)
C3—C4—C5—S1	2.4 (5)	C9'—N1—C7'—O5	174.8 (10)
C2—S1—C5—C4	−4.4 (3)	C10'—N1—C7'—C8'	173.1 (12)
C2—S1—C5—C6	−176.4 (3)	C10—N1—C7'—C8'	−4.8 (11)
Zn1^v^—O4—C6—O3	−5.8 (5)	C7—N1—C7'—C8'	175.2 (15)
Zn1^v^—O4—C6—C5	171.8 (2)	C9—N1—C7'—C8'	164 (2)
Zn1^iv^—O3—C6—O4	4.4 (6)	C9'—N1—C7'—C8'	−5.0 (14)
Zn1^iv^—O3—C6—C5	−173.1 (2)		

Symmetry codes: (i) −*x*+1, −*y*+1, −*z*+1; (ii) −*x*+3/2, *y*+1/2, −*z*+1/2; (iii) *x*−1/2, −*y*+1/2, *z*+1/2; (iv) *x*+1/2, −*y*+1/2, *z*−1/2; (v) −*x*+3/2, *y*−1/2, −*z*+1/2.
